# Comparative evaluation of mechanical and biological prostheses in patients with aortic stenosis

**DOI:** 10.1093/icvts/ivaf091

**Published:** 2025-04-10

**Authors:** Juan Andres Montero, Federica Venturino, Santiago Cubas, Sofía Rodríguez, Maximiliano Hernández, Carolina Sosa, Maximiliano Rodríguez, Daniel Brusich, Victor Dayan

**Affiliations:** Centro Cardiovascular Universitario, Hospital de Clínicas, Universidad de la República, Montevideo, Uruguay; Centro Cardiovascular Universitario, Hospital de Clínicas, Universidad de la República, Montevideo, Uruguay; Centro Cardiovascular Universitario, Hospital de Clínicas, Universidad de la República, Montevideo, Uruguay; Centro Cardiovascular Universitario, Hospital de Clínicas, Universidad de la República, Montevideo, Uruguay; Centro Cardiovascular Universitario, Hospital de Clínicas, Universidad de la República, Montevideo, Uruguay; Centro Cardiovascular Universitario, Hospital de Clínicas, Universidad de la República, Montevideo, Uruguay; Centro Cardiovascular Universitario, Hospital de Clínicas, Universidad de la República, Montevideo, Uruguay; Centro Cardiovascular Universitario, Hospital de Clínicas, Universidad de la República, Montevideo, Uruguay; Centro Cardiovascular Universitario, Hospital de Clínicas, Universidad de la República, Montevideo, Uruguay

**Keywords:** aortic valve replacement, mechanical prostheses, bioprostheses, survival

## Abstract

**OBJECTIVES:**

The commonly accepted aortic valve prostheses have been either mechanical or biological. Each type has its advantages and disadvantages, with age being the most widely accepted variable to determine the best option. There is, however, a range between 60 and 70 years where an individualized approach is required.

**METHODS:**

This is a retrospective study. The primary outcome was overall survival based on the type of prosthesis used, stratified by effect modifiers. Association between prosthesis type and mortality rate was evaluated using the incidence rate ratio. Secondary outcomes included cardiovascular survival, postoperative mortality and complications, adjusted for age. Cox regression analysis was performed to account for confounders. Variation in the hazard ratio for death by age was explored by fitting a restricted cubic spline to the interaction between age and valve type. We included all adult patients who underwent surgical aortic valve replacement for severe stenosis in Uruguay from 2011 to 2021. A total of 3944 patients were enrolled; 1708 were females. Median follow-up time was 4.5 years.

**RESULTS:**

Bioprostheses (BP) were associated with higher mortality in males and in patients without statins. When mortality rate was stratified by age, BP were associated with a higher risk in patients younger than 60 and a lower risk in the 70–79 age group.

**CONCLUSIONS:**

BP are associated with worse survival in male patients and in the <60-year-old age group. Gender and statins should be considered when deciding the prosthesis for patients in the 60–69 age group. When the relative survival benefit of BP was analysed, 70 years was identified as the threshold at which their benefit became evident compared to mechanical prostheses.

## Graphical Abstract

**Figure:**
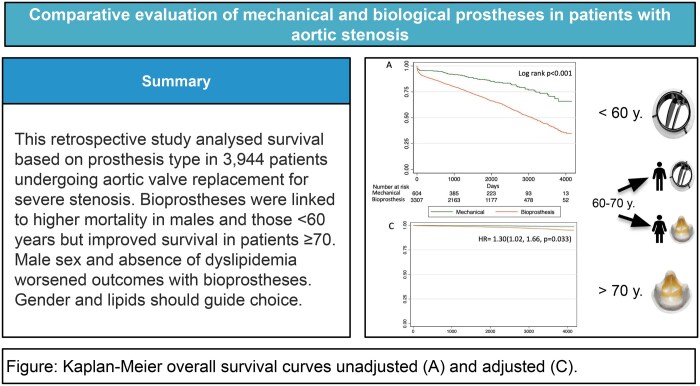

## INTRODUCTION

The effective treatment for severe aortic valve disease remains surgical aortic valve replacement (SAVR) or transcatheter aortic valve implantation (TAVI). To date, the commonly accepted aortic valve prosthesis are the mechanical prostheses (MP) or the BP. Each has its advantages and disadvantages, with age currently being the most frequently accepted clinical variable to define the best option. MP is likely to be recommended for younger patients (<50–60 years), while BP is preferred for older patients (>65 years) [1, 2]. There is an age range between 50 and 65 years, according to the guidelines of the American College of Cardiology and the American Heart Association (ACC/AHA), or between 60 and 65 years as per the guidelines of the European Society of Cardiology and the European Association of Cardiothoracic Surgery (ESC/EACTS), where an individualized approach is recommended. In this age group, the only published randomized controlled trial found no differences in survival, with a higher incidence of prosthetic failure and reoperation in the BP group [3]. A recent meta-analysis of primarily retrospective studies found that MP were associated with better survival in this age group [4]. Considering the increasing number of TAVI procedures performed in younger patients (<70 years), it is imperative to confirm the superiority of BP (or not) in comparison to MP in this age group, as well as the predictors that may favour one prosthesis over the other for this group. Our objective was to evaluate survival in a national cohort of patients who required SAVR due to aortic stenosis (AS) using either MP or BP.

## METHODS

### Ethical statement

The Ethics Review Board of the Hospital de Clínicas (approval number 6220001) approved the study on the 22nd of March 2022, and the requirement for informed consent was waived due to the retrospective nature of the research.

This is a retrospective study using data from Uruguay, sourced from the National Resource Fund (FNR) that is the national entity that fully covers 100% of cardiac surgical procedures in Uruguay. Data submission to the FNR is mandatory to obtain financial approval for each procedure.

### Patients

All patients who underwent SAVR for AS in Uruguay between 2011 and 2021 were included (type and model of prostheses used in Supplementary Material, Table S3). Exclusion criteria were urgent or emergency surgery, endocarditis, moderate AS, severe aortic reguritation, previous cardiac surgery, concomitant mitral valve or ascending aorta replacement.

Preoperative, intraoperative and immediate postoperative data were extracted from the FNR. Expected mortality was assessed using EuroSCORE I (logistic model). Calculation of EuroSCORE II was not feasible as the study included patients from before EuroSCORE II was established, and the registry lacks the necessary data for its calculation. Vital status and cause of death were obtained from the Department of Epidemiology of the Ministry of Health.

### Objectives

The primary outcome was overall survival based on the type of prostheses used, stratified by effect modifiers. The association between prostheses type and mortality rate was evaluated using the incidence rate ratio (IRR). Secondary objectives included cardiovascular survival defined as time from surgery until death due to cardiovascular causes such as acute myocardial infarction, sudden cardiac death or heart failure and postoperative outcomes (postoperative mortality, surgical re-exploration, infections, stroke, atrial fibrillation, atrioventricular block, patient-prosthesis mismatch and acute renal failure) adjusted by age.

Due to a large percentage of patients with BP who received concurrent coronary artery bypass graft (CABG) (36%), a final model for hazard ratio (HR) for overall survival in patients with BP after multivariate adjustment excluding patients who underwent CABG can be found in Supplementary Material (Table S4).

### Statistics

Normality of continuous variables was assessed by the Shapiro test. Continuous data are expressed as mean ± SD, while categorical data are expressed as absolute values and percentages. Continuous variables such as age, creatinine levels, left ventricular ejection fraction (LVEF) and EuroSCORE were categorized as follows: age (<60, 60–69, 70–79, >80 years), creatinine levels (<1.2 and >1.2 mg/dl), LVEF (>50%, 50–30%, <30%) and EuroSCORE (<5%, 5–15%, >15%).

Preoperative variables were compared between the groups to identify potential confounders using the chi-squared test (for categorical variables) and Student’s t-test (for continuous variables). Potential confounders were then evaluated using univariate Cox regression analysis to determine their role as confounding factors. Univariate confounding and effect modification were assessed through Mantel-Haenszel stratification, and multivariate adjustment was performed using Cox regression. Interaction in the multivariate Cox regression model was evaluated using the likelihood ratio test for variables identified as modifiers in the univariate analysis. If interaction was present, the interaction term was included in the final model.

Long-term mortality was assessed as the mortality rate, with the numerator being the number of events (deaths) and the denominator being person-days of follow-up. The association between prosthesis type and mortality rate was evaluated using the IRR (Supplementary Material, Table S1). The IRR and its 95% confidence interval (CI) were calculated using the Mantel-Haenszel method. Considering that the age group of 60–70 years is recommended for either bioprosthesis or mechanical valve, the IRR was also calculated for each age group, and a subgroup analysis was performed for patients aged 60–70 years. Interaction was assessed using the chi-squared test. Cases with missing information were excluded in the multivariate analysis.

Survival was analysed using the Kaplan–Meier method, and comparisons between groups were made using the log-rank test.

Postoperative outcomes were compared between groups and adjusted for age groups using logistic regression.

The variation in the mortality risk index between MP and BP by age was explored by adjusting a restricted cubic spline to the interaction between age and valve type, using five knots.

## RESULTS

A total of 3944 patients who underwent SAVR in Uruguay between 2011 and 2021 were included in the study. The mean age of the cohort was 71 (SD: 9.4) years, most of them were women (43.3% vs 56.7%, *P* < 0.001), and the mean EuroSCORE I was 6.9% (7.47% in BP vs 4.02% in MP, *P* < 0.001). Patients receiving a BP were older on average (73.5 vs 57.5 years, *P* < 0.001) and had a higher prevalence of female sex (44.6% vs 36.3%, *P* < 0.001), diabetes (25% vs 18.7%, *P* < 0.001), statins (44.4% vs 32.9%, *P* < 0.001), hypertension (81.6% vs 64.3%, *P* < 0.001), elevated creatinine levels (>1.2 mg/dl) (24.5% vs 18.7%, *P* < 0.001) and concomitant coronary surgery (36% vs 20%, *P* < 0.001) (Table 1). The predicted mortality was higher in patients receiving BP (7.47 (SD: 4.30) vs 4.02 (SD: 3.38), *P* < 0.001).

**Table 1:** Baseline characteristics of the included patients (*n* = 3944)

	BP (*n* 3333)	MP (*n* 611)	Total (*n* 3944)	*P*
Mean age (SD)	73.5 (6.8)	57.5 (10.3)	71.0 (9.4)	<0.001
Age (%)				<0.001
<60	74 (2.2)	354 (57.9)	428 (10.85)	
60–69	831 (24.9)	201 (32.9)	1032 (26.2)	
70–79	1775 (53.3)	45 (7.4)	1820 (46.1)	
≥80	653 (19.6)	11 (1.8)	664 (16.8)	
Female (%)	1486 (44.6)	222 (36.3)	1708 (43.3)	<0.001
Hypertension (%)	2721 (81.6)	393 (64.3)	3114 (79.0)	<0.001
Diabetes (%)	834 (25.0)	114 (18.7)	948 (24.0)	<0.001
Smoker (%)	763 (22.9)	150 (25.6)	913 (23.2)	0.37
Obesity (%)	628 (18.8)	70 (11.4)	698 (17.7)	
Creatinine mg/dl (SD)	1.08 (0.70)	1.06 (0.86)	1.08 (0.73)	0.43
Statins (%)	1481 (44.4)	201 (32.9)	1682 (42.7)	<0.001
Previous infarction (%)	69 (2.1)	7 (1.2)	76 (1.9)	0.12
Atrial fibrillation (%)	81 (2.4)	13 (2.1)	94 (2.4)	0.65
NYHA III/IV (%)	805 (28.6)	131 (26.3)	936 (28.3)	0.62
LVEF (SD)	57.5 (10.6)	58.0 (10.4)	57.6 (10.5)	0.28
Concomitant CABG (%)	1201 (36.0)	122 (20.0)	1323 (33.5)	<0.001
EuroSCORE I (SD)	7.47 (4.30)	4.02 (3.38)	6.9 (4.4)	<0.001

NYHA: New York Heart Association; SD: standard deviation.

### Primary objective

**Figure 1:**
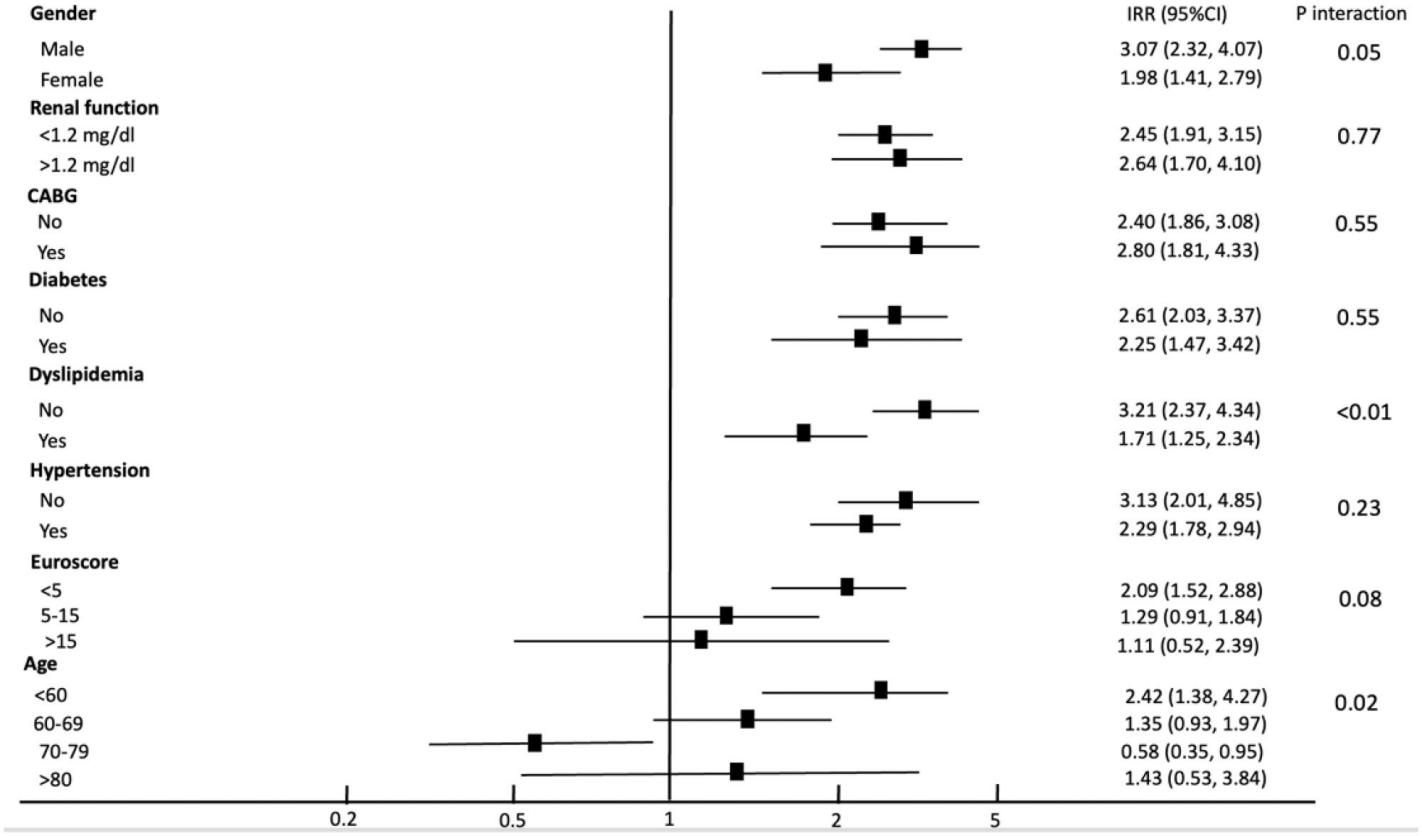
Forest plot for mortality incidence rate ratio for bioprostheses with adjustment for confounders and stratum-specific associations (*n* = 3944)

The unadjusted IRR showed a higher risk in patients receiving BP (IRR = 2.57; 95% CI: 2.07–3.20, *P* < 0.001). Univariate adjustment revealed that age (*P* for interaction = 0.02), sex (*P* for interaction = 0.05) and statins (*P* for interaction < 0.01) were effect modifiers of the impact of BP on mortality rate (Fig. 1). When stratifying mortality rates by age group, BP was associated with higher risk in patients under 60 years (IRR = 2.42; 95% CI: 1.38–4.27, *P* for interaction = 0.02) and lower risk in patients aged 70–79 years (IRR = 0.58; 95% CI: 0.35–0.95, *P* for interaction = 0.02) (Fig. 1). No significant differences were observed in the 60–69 or >80 age groups. Similarly, the impact of BP on mortality was greater in males (IRR = 3.07; 95% CI: 2.32–4.07) compared to females (IRR = 1.98; 95% CI: 1.41–2.79, *P* for interaction = 0.05) and was higher in patients without statins (IRR = 3.21; 95% CI: 2.37–4.34) compared to those with statins (IRR = 1.71; 95% CI: 1.25–2.34, *P* for interaction = 0.02).

**Figure 2:**
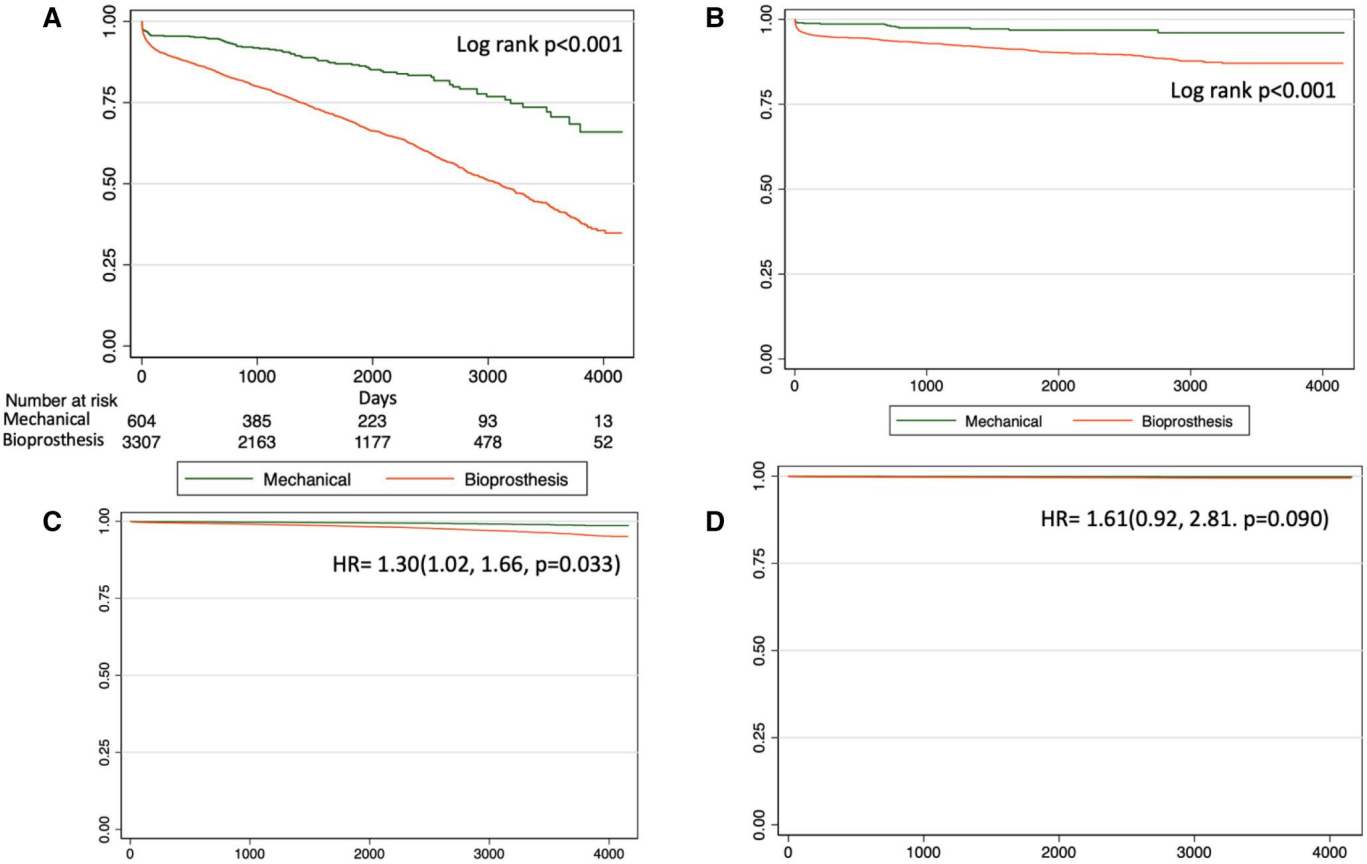
Kaplan–Meier survival curves. Overall (**A**) unadjusted and (**C**) adjusted survival. Cardiovascular (**B**) unadjusted and (**D**) adjusted survival

Adjusted and unadjusted survival was lower in patients receiving BP (Fig. 2).

Univariate predictors of survival included age, sex, creatinine levels, concomitant coronary surgery, EuroSCORE, BP, statins, diabetes and hypertension (Supplementary Material, Table S2).

After multivariate regression and interaction analysis, BP use was an independent predictor of overall survival in male patients (HR = 1.45; 95% CI: 1.06–1.98, *P* = 0.018) but not in females (HR = 0.90; 95% CI: 0.63–1.30). Additionally, its use was significantly associated with worse outcomes in patients under 60 years (HR = 2.51; 95% CI: 1.42–4.13) and better outcomes in the 70–79 age group (HR = 0.65; 95% CI: 0.39–1.06) (Table 2). Although no significant interaction was observed after multivariate adjustment for statins, a strong negative effect on survival was noted in patients without statins.

**Table 2:** Final model for HR for overall survival in patients with bioprostheses after multivariate adjustment (*n* = 3944)

Variable	Multivariate	*P*	*P* for interaction
Bioprosthesis			0.04
Male	1.45 (1.06, 1.98)	0.02	
Female	0.90 (0.63, 1.30)	0.59	
<60 year old	2.51 (1.42, 4.13)	< 0.001	0.01
60–69 year old	1.19 (0.82, 0.58)	0.36	
70–79 year old	0.65 (0.39, 1.06)	0.09	
>80 year old	1.75 (0.63, 4.71)	0.27	
Age	1.02 (1.01, 1.03)	<0.001	
Creatinine	1.09 (1.04, 1.16)	<0.001	
Statins	1.16 (1.04, 1.30)	<0.001	0.16
Diabetes	1.29 (1.14, 1.47)	<0.001	
Hypertension	1.16 (1.00, 1.35)	0.05	
CABG	1.11 (0.99, 1.24)	0.07	

**Figure 3:**
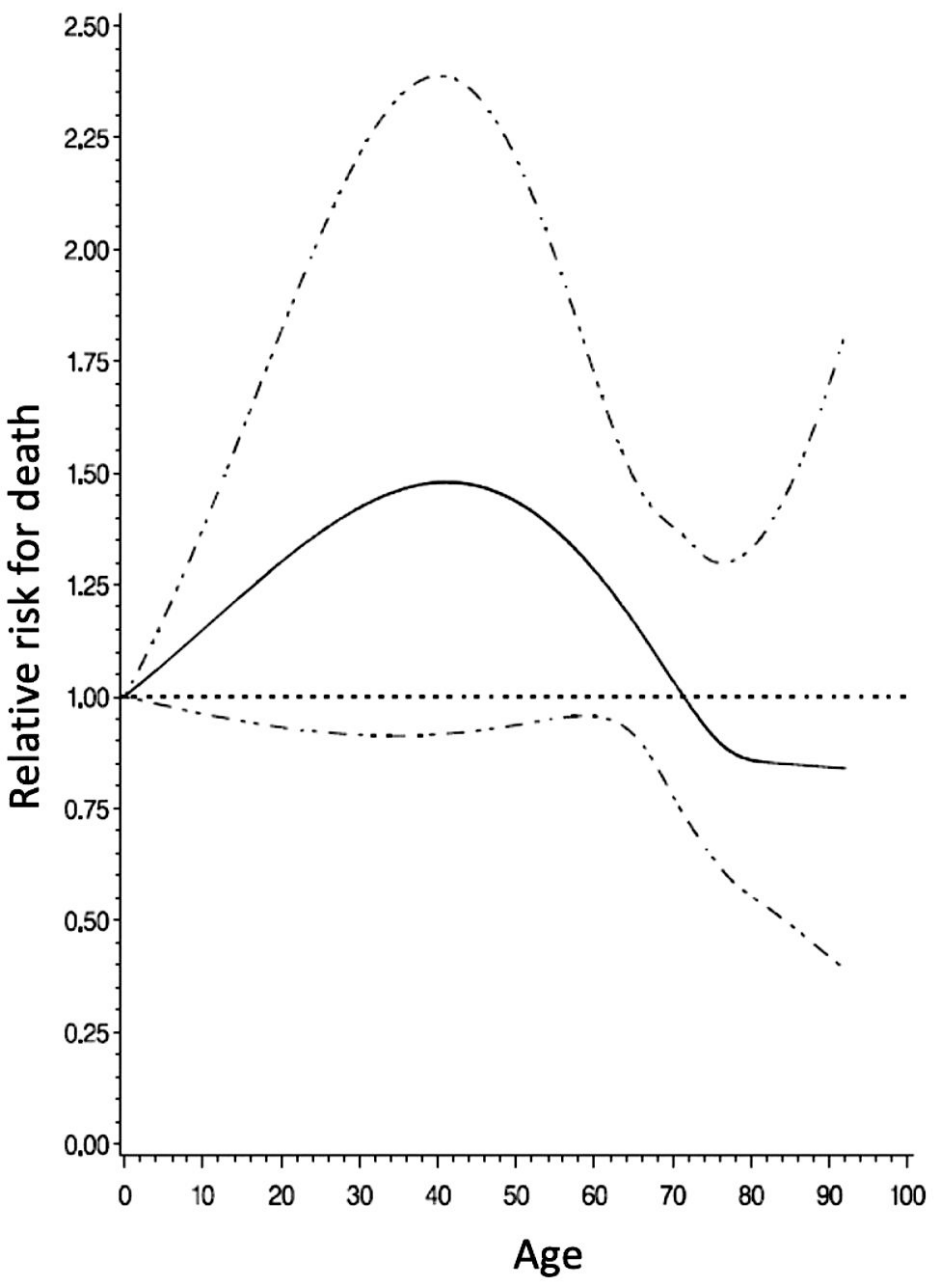
Hazard ratio of death and 95% confidence intervals (dashed lines) by age

When analysing the relative risk of survival benefit with BP by increasing age, 70 years emerged as the threshold at which BP demonstrated a survival benefit over MP (Fig. 3).

### Patients aged 60–69 years

Subgroup analysis of patients aged 60–69 years showed no interaction in any analysed subgroups. However, male sex (IRR = 1.67; 95% CI: 1.04–2.70) and absence of statins (IRR = 1.83; 95% CI: 1.06–3.18) were significantly associated with worse outcomes with BP (Supplementary Material, Fig. S1).

### Secondary objectives

Unadjusted cardiovascular survival was lower in patients receiving BP, but no differences were found after adjusting for confounders (Fig. 2).

There were no differences in postoperative outcomes except for an increased risk of postoperative atrial fibrillation with BP (OR = 1.34; 95% CI: 1.05–1.71, *P* = 0.02) (Table 3).

**Table 3:** Postoperative outcomes

	Bioprosthesis (3333)	Mechanical (611)	OR (95%CI)	P*
Operative mortality	198 (5.9)	23 (3.8)	0.86 (0.51, 1.43)	0.55
Surgical re-exploration	181 (5.4)	22 (3.6)	1.42 (0.84, 2.39)	0.19
Postoperative infections	475 (14.3)	63 (10.3)	1.01 (0.72, 1.40)	0.98
Stroke	83 (2.49)	9 (1.47)	1.55 (0.70, 3.44)	0.28
Atrial fibrillation	1238 (37.1)	122 (20.0)	1.34 (1.05, 1.71)	0.02
AV block	239 (7.2)	33 (5.4)	0.98 (0.63, 1.52)	0.93
PPM	112 (3.4)	15 (2.5)	1.09 (0.58, 2.07)	0.79
Acute renal failure	417 (12.5)	43 (7.0)	0.94 (0.66, 1.34)	0.74

AV: atrioventricular; PPM: permanent pacemaker; P*: P value for interaction.

## DISCUSSION

After multivariate analyses, BP were associated with worse survival in male patients and in those under 60 years of age. A trend towards improved survival was observed in the 70–79 age group. The significant association between survival and BP use in patients without statins, as well as the influence of gender, should be considered when deciding on the type of prosthesis for patients aged 60–69 years. This was discussed in depth in a companion article published in 2024 by Dayan *et al.* about medium-term survival of patients with aortic prosthesis at the sixth decade of life [5]. There is no clear age threshold at which the ACC/AHA or ESC/EACTS guidelines decisively recommend BP over MP, or vice versa. Current guidelines suggest that patients under 50 years of age may benefit from MP, while those over 65 years are generally recommended for BP use [1, 2]. However, studies on intermediate age groups (50–70 years) have shown conflicting results. Chiang *et al.* [6], in a 10-year retrospective follow-up of patients undergoing SAVR in New York State, reported no differences in long-term mortality in this age group. Similarly, Attia *et al.* [7], using data from the Cleveland Clinic, found no differences in long-term survival between valve types in this age group. Moreover, they reported no survival differences in patients requiring reintervention. However, Attia *et al.*’s findings may have limited external validity and should be interpreted with caution when applied to cardiac surgery centres with lower surgical volumes.

In contrast, other studies have reported better survival with MP in this age group. For instance, Brown *et al.* [8] in the USA and Glaser *et al.* [9] in Sweden demonstrated superior survival rates with MP in patients aged 50–70 years. These discrepancies highlight the need for individualized decision-making based on patient characteristics, local expertise and institutional factors. Most data comparing BP and MP originate from the USA or Europe. Factors such as anticoagulation management and technical expertise at cardiac surgery centres significantly influence the relative benefits of each valve type. Thus, evidence from centres outside these regions is crucial to gaining a global perspective and improving the external validity of available data.

Our national data, derived from a Latin American country, do not align with the findings of Goldstone *et al.* [10] in California. Goldstone *et al.* reported that the survival benefit of MP persisted only up to age 55. In contrast, we identified age 70 as the turning point where the benefit of MP was surpassed by BP.

The primary disadvantage of BP is degeneration and the need for reintervention. However, if severe degeneration is detected early and reoperations are successfully performed, the disadvantages of BP are mitigated, potentially shifting the age threshold for benefit towards younger patients.

Cardiac surgery centres in Uruguay, representing Latin American countries, likely differ from those in the USA in terms of delayed diagnosis and potentially worse outcomes following reinterventions. These factors may explain the observed benefit of MP up to age 70 in our population.

Clearly, the optimal choice of prosthesis for patients aged 50 to 70 is not absolute. Identifying subgroups of patients who may benefit from either valve type is essential. Gender is a particularly important subgroup that has been insufficiently studied in the literature.

Kulik *et al.* [11] found better outcomes with BP in female patients, possibly due to a reduced need for reinterventions. Similarly, a Finnish nationwide study [12] demonstrated a lower reintervention rate in women. Hammermeister *et al.* [13] in a randomized trial of male patients, reported higher long-term mortality with BP. Other studies have shown that gender is a significant confounder in the relative benefits of MP [14].

Although high-dose statin therapy has not succeeded in halting the calcification of native aortic valves [15], limited evidence exists regarding their benefit in BP. Antonini-Canterin *et al.* [16] demonstrated an association between statin use and a reduction in the progression of BP degeneration. Additionally, rosuvastatin has been shown in animal models to decrease BP calcification [17]. In our cohort, patients with dyslipidaemia who received BP exhibited better outcomes. While medication use was not a variable in our database, it is likely that these patients were on statins, and the observed benefit may be attributable to their reported role in slowing BP degeneration. Further randomized controlled trials are necessary to test this association. This difference may be related to the inflammatory response and haemodynamic stress associated with biological valves, also knowing that the age and number of comorbidities that these patients had at the time of surgery was greater, these results were similar to those found by Rikhard Björn *et al.* [18].

We know that these results may vary today, given the advances in valve-in-valve TAVI (ViV-TAVI), which make it necessary to choose a bioprosthesis for patients at high risk of reintervention, thereby increasing their survival. A recent study by Danial Ahmad *et al.* [19] retrospectively compared patients who underwent ViV-TAVI and native valve TAVI. Of 3532 patients, 198 (5.6%) underwent ViV-TAVI. The ViV-TAVI group had a higher incidence of major vascular complications (2.5% vs 0.8%, *P* = 0.008), a lower rate of permanent pacemaker placement (2.5% vs 8.1%, *P* = 0.004) and a comparable incidence of stroke (2.5% vs 2.4%, *P* = 0.911). In a multivariate Cox regression analysis, ViV-TAVI was associated with a reduced risk of death (HR 0.68 [0.5 to 0.9], *P* = 0.02). Therefore, ViV-TAVI appears to be a feasible and safe strategy for high-risk patients with bioprosthetic valve failure, potentially influencing survival rates and prosthesis selection in this patient group. As well as with new generation biological valves that promise greater durability, thanks to an anticalcification coating process on its leaflets, it ensures better haemodynamics and improvements in tissue preservation processes, which could translate into greater durability and better clinical performance. In addition, it incorporates new technology, which facilitates future ViV procedures in patients who may require reinterventions. The COMMENCE trial by Bavaria *et al.* [20] published in 2023 confirms these hypotheses after following patients with new generation RESILIA tissue valves for 5 years.

## CONCLUSION

Our findings confirm that gender strongly influences outcomes after SAVR and should be carefully considered. Our data suggest that male patients in the 60–70 year-old age group likely benefit more from MP, while female patients may achieve favourable outcomes with either strategy.

### Limitations

As a retrospective study, selection bias is the primary limitation. To account for measured confounders, we performed Cox regression analyses. However, we acknowledge the potential influence of unmeasured confounders on the results.

We relied on the national database to extract outcome variables. The follow-up of postoperative outcomes and cross-referencing with other national databases is suboptimal. Consequently, we were unable to evaluate critical outcomes such as readmissions, reoperations, strokes or bleeding events.

## Supplementary Material

ivaf091_Supplementary_Data

## Data Availability

The data underlying this article will be shared upon reasonable request to the corresponding author.

## Notes

Presented at the 2024 STS/EACTS/LACES Latin America Cardiovascular Surgery Conference, Buenos Aires, Argentina.

## ABBREVIATIONS

AS: Aortic stenosis
BP: Bioprostheses
CI: Confidence interval
FNR: National Resource Fund
IRR: Incidence rate ratio
LVEF: Left ventricular ejection fraction
MP: Mechanical prostheses
SAVR: Surgical aortic valve replacement
TAVI: Transaortic aortic valve implantation
