# Supplementary material for: Comparative evaluation of mechanical and biological prostheses in patients with aortic stenosis
Source: Interdiscip Cardiovasc Thorac Surg. 2025 Apr 10;40(5):ivaf091. doi: 10.1093/icvts/ivaf091 (PMC12055754; doi:10.1093/icvts/ivaf091)

Supplementary Table 1. Mortality rate stratified by age group.

| Prosthesis | Deaths | Person.days (per 100000) | Rate (95%CI) | Rate ratio | p |
| --- | --- | --- | --- | --- | --- |
| **Overall** | | | | | |
| Mechanical | 87 | 9.82 | 8.86 (7.18, 10.93) | 1 (Reference) | <0.001 |
| Bioprosthesis | 1211 | 53.11 | 22.80 (21.55, 24.12) | 2.57 (2.07, 3.20) |  |
| **<****60 year old** | | | | | |
| Mechanical | 36 | 6.50 | 5.53 (3.99, 7.67) | 1 (Reference) | 0.015 |
| Bioprosthesis | 18 | 13.41 | 13.41 (8.45, 21.28) | 2.42 (1.38, 4.27) |  |
| **60-69 year old** | | | | | |
| Mechanical | 31 | 2.76 | 11.24 (7.91, 15.99) | 1 (Reference) | 0.113 |
| Bioprosthesis | 234 | 15.39 | 15.20 (13.37, 17.28) | 1.35 (0.93, 1.97) |  |
| **70-79 year old** | | | | | |
| Mechanical | 16 | 0.39 | 41.30 (25.30, 67.42) | 1 (Reference) | 0.029 |
| Bioprosthesis | 654 | 27.33 | 23.93 (22.16, 25.83) | 0.58 (0.35, 0.95) |  |
| **>****80 year old** | | | | | |
| Mechanical | 4 | 0.17 | 23.52 (8.83, 62.67) | 1 (Reference) |  |
| Bioprosthesis | 305 | 9.04 | 33.71 (30.13, 37.71) | 1.43 (0.53, 3.84) |  |

Supplementary Table 2. Univariate predictors of survival.

| Variable | Univariate (HR95%CI) | p |
| --- | --- | --- |
| Bioprosthesis | 2.58 (2.07, 3.21) | <0.001 |
| Age |  | <0.001 |
| <60 | 1 |  |
| 60-69 | 2.11 (1.57, 2.82) | <0.001 |
| 70-79 | 3.53 (2.68, 4.66) | <0.001 |
| >80 | 4.93 (3.69, 6.58) | <0.001 |
| Female | 0.89 (0.80, 1.00) | 0.045 |
| Creatinine >1.2 | 1.67 (1.49, 1.88) | <0.001 |
| Statins | 1.39 (1.24, 1.55) | <0.001 |
| Diabetes | 1.53 (1.36, 1.72) | <0.001 |
| Hypertension | 1.35 (1.17, 1.56) | <0.001 |
| CABG | 1.31 (1.16, 1.45) | <0.001 |
| EuroSCORE I | 2.34 (2.11, 2.61) | <0.001 |

Supplementary Table 3. Type and model of prostheses used.

| **Valve** | **n** | **%** |
| --- | --- | --- |
| Bioprosthesis - ST. JUDE MEDICAL - EPIC | 954 | 24.2 |
| Bioprosthesis - SORIN - MITROFLOW AORTIC | 693 | 17.6 |
| Bioprosthesis - MEDTRONIC - MOSAIC | 406 | 10.3 |
| Bioprosthesis - SORIN - CROWN PRT AORTIC | 383 | 9.7 |
| Bioprosthesis - MEDTRONIC - HANCOCK II | 262 | 6.6 |
| Mechanical - SORIN - BICARBON SLIMLINE | 236 | 6.0 |
| Mechanical - ST. JUDE MEDICAL - REGENT | 235 | 6.0 |
| Bioprosthesis - EDWARDS - PERIMOUNT | 185 | 4.7 |
| Bioprosthesis - MEDTRONIC - HANCOCK II | 155 | 3.9 |
| Bioprosthesis - LABCOR - DOKIMOS PLUS | 100 | 2.5 |
| Bioprosthesis - BIOMEDICA - PROSTHETIC VALVE | 93 | 2.4 |
| Others | 58 | 1.5 |
| Bioprosthesis - ST. JUDE MEDICAL - TRIFECTA | 51 | 1.3 |
| Mechanical - CRYOLIFE - ON-X AORTIC | 43 | 1.1 |
| Bioprosthesis - SORIN - PERCEVAL S AORTIC | 35 | 0.9 |
| Mechanical - ATS MEDICAL - AP AORTIC | 23 | 0.6 |
| Bioprosthesis - MEDTRONIC - AVALUS | 16 | 0.4 |
| Mechanical - MEDTRONIC - BIVALVA ADVANCED | 16 | 0.4 |

Supplementary table 4. Final model for HR for overall survival in patients with bioprostheses after multivariate adjustment excluding patients who underwent CABG (n=2600).

| **Variable** | **Multivariate** | **p** |
| --- | --- | --- |
| Bioprosthesis | 1.14 (0.84, 1.53) | 0.40 |
| Female | 0.90 (0.78, 1.04) | 0.15 |
| Statins | 1.10 (0.95, 1.28) | 0.22 |
| Age | 1.03 (1.02, 1.05) | <0.001 |
| Creatinine | 1.08 (1.02, 1.15) | 0.012 |
| Diabetes | 1.29 (1.08, 1.53) | 0,004 |
| Hypertension | 1.11 (0.92, 1.34) | 0.28 |
| CABG | 79.76 (21.88, 290.77) | <0.001 |

CABG: coronary artery bypass grafts.

Supplementary Figure 1. Forest plot for mortality incidence rate ratio for bioprostheses with adjustment for confounders and stratum-specific associations in 60-69 year old patients (n=1032).


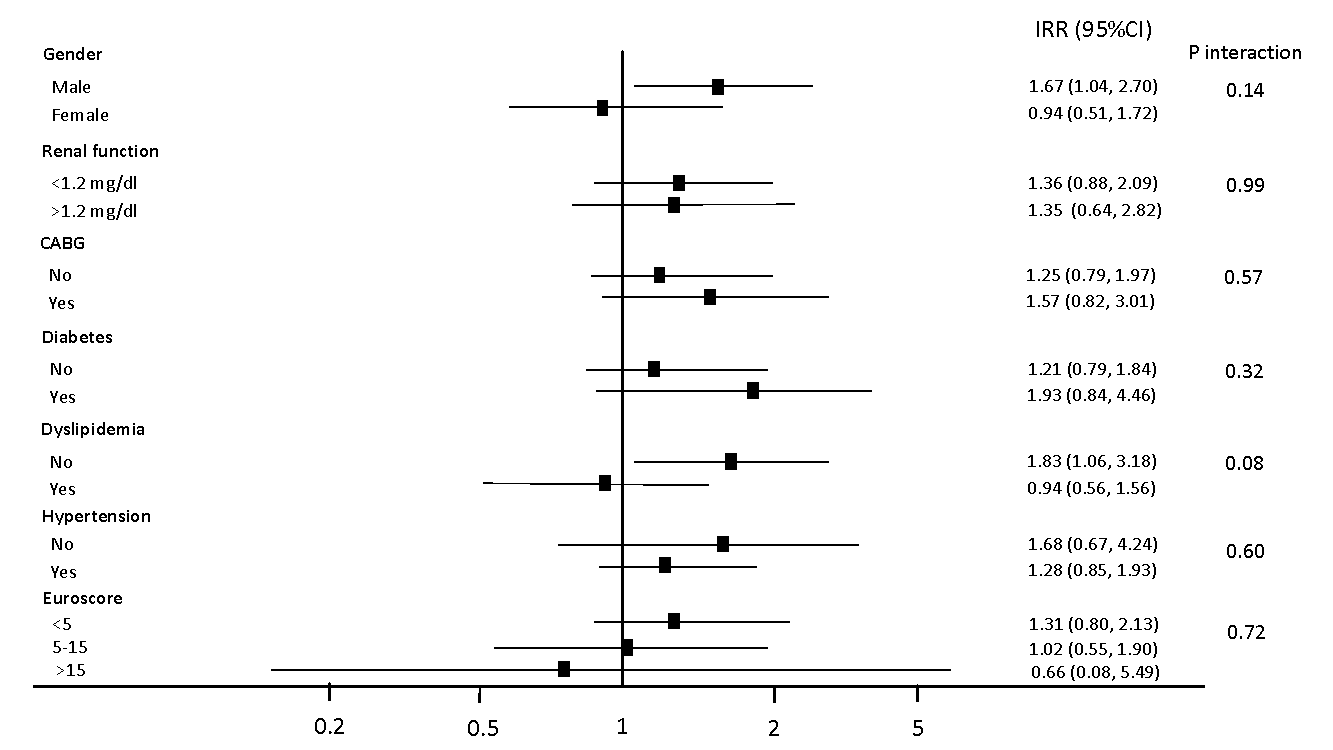

Supplement: ivaf091_Supplementary_Data [file ivaf091_supplementary_data.zip › Supplementary_material_(3).docx]
